# Constitutivism and Transcendental Practical Philosophy: How to Pull the Rabbit Out of the Hat

**DOI:** 10.1007/s11406-016-9746-3

**Published:** 2016-09-16

**Authors:** Sorin Baiasu

**Affiliations:** Philosophy Programme, School of Politics, Philosophy, International Relations and Environment, Keele University, Keele, ST5 5BG UK

**Keywords:** Immanuel Kant, Christine Korsgaard, Michael Smith, Constitutivism, Constructivism, Transcendental argument, Anaytic/synthetic, Metaphysics

## Abstract

Constitutivism aims to justify substantial normative standards as constitutive of practical reason. In this way, it can defend the constructivist commitment to avoiding realism and anti-realism in normative disciplines. This metaphysical debate is the perspective from which the nature of the constitutivist justification is usually discussed. In this paper, I focus on a related, but distinct, debate. My concern will not be whether the substantial normative claims asserted by the constructivist have some elements, which are not constructed, but real, given independently from us; instead, my concern will be more narrowly epistemic – whether those claims can be derived from premises, which are normatively less substantial than the normative conclusions themselves. I focus on Korsgaard’s transcendental articulation of the constitutivist argument. I conclude that more work would need to be done, in order for this argument to function as intended.

## Introduction

Constitutivism seems to be one of the more promising versions of constructivism.^1^ It aims to justify substantial normative standards as constitutive of practical reason. In this way, it can defend the constructivist commitment to avoiding realism and anti-realism in normative disciplines, such as ethics. Standards of action are not already given, independently from us, but they are justified as constitutive of practical reason; yet, they are not the result of arbitrary decisions, but are conditions, which make possible agency. Nevertheless, the nature of the justification, which constitutivism can offer in support of the normative standards it puts forward, is usually discussed from the perspective of the debate between realism and anti-realism, the debate between the view that there are normative standards independently from agents and the view that normative standards are created by the (arbitrary) decisions of agents.^2^

In this paper, I would like to focus on a related, but distinct, debate. My concern will not be whether the substantial normative claims asserted by the constructivist have some elements, which are not constructed, but real, given independently from us; instead, my concern will be more narrowly epistemic – whether those claims can be derived from premises, which are normatively less substantial than the normative conclusions themselves. Whether the premises or conclusions are constructed or independent from construction is, in this context, almost irrelevant; what is relevant is the extent to which an argument can enrich normatively its premises, so that we would start from some premises, which are normatively weaker, and end up with conclusions, which are normatively stronger.

Assuming that such a feat turns out to be possible, we could deal much more easily with sceptical arguments.^3^ It is sufficient to start from premises sufficiently weak to be accepted by the sceptic and to derive in a justified way normatively stronger conclusions, in order to be able then to reject the sceptical position about the stronger, more substantial normative claims. I have said that my concern is with an issue, which is distinct from the debate between realism, anti-realism and constructivism, but related to this debate. This relation is easily noticeable in a particular case. Thus, if we start with merely descriptive premises and succeed in deriving normative claims through a constitutivist argument, then those normative claims constructed through the justification provided by the argument could no longer be suspected of secretly relying on some realist ground, on some normative component smuggled in, in the premises of the argument. In other words, a successful constitutivist argument could help constructivists show that it is possible to justify substantial normative claims in a non-arbitrary manner and without realist premises. Yet, as already mentioned, this is not my concern here.

Now, one of the prominent constitutivist positions in the literature is Christine Korsgaard’s and, in this paper, I will focus on its articulation in her *The Sources of Normativity*. (1996)^4^ The articulation of the constitutivist position in this text is particularly important, since it suggests the kind of argument one would need, in order to move from normatively weaker premises to normatively substantial conclusions. Nevertheless, while a promising approach to this issue is suggested by Korsgaard, this suggestion is not explored further in her texts.^5^ In this paper, I plan to examine this approach more closely and to suggest how it can work successfully.

In the next section, I will consider one recent objection to Korsgaard’s constitutivism, an objection according to which, far from beginning from slender premises, she would start from a normatively substantial account of our essential identity and would then attempt to derive from it robust normative claims. I argue that this objection relies on an inaccurate interpretation of her position and, after a clarification, in Section 3, of some elements of the conceptual framework of the discussion, I present (in Section 4) what I take to be an accurate interpretation. Section 5 reconstructs the crucial argument of Korsgaard’s constitutivism and Section 6 evaluates it. I conclude that more work would need to be done, in order for Korsgaard’s argument to function as intended. One reason why this additional work might not seem a welcome complement to her position is due to an important implication: in order for Korsgaard’s constitutivism to work, she would also need to adopt a metaphysical position she might not be keen to embrace. But let us begin with the objection to Korsgaard.

## One Objection to Korsgaard’s Constitutivism

According to Michael Smith,

Constitutivism is the view that we can derive a substantive account of normative reasons for action – perhaps a Kantian account, perhaps a hedonistic account, perhaps a desire-fulfilment account, this is up for grabs – from abstract premises about the nature of action and agency. (2015: 187)

Following this definition, therefore, constitutivism claims that we can get to a substantial account of normative reasons for action from an account of the nature of action and agency. Going from a view of action in general to a view of reasons for or against the performance of particular actions seems to involve the creation of something ex nihilo. As Eric Wiland notes, in relation to constitutivist arguments, “[t]hose who claim to extract reasons out of agency remind us of those who claim to pull rabbits out of hats”. (Wiland 2012: 141)^6^

Smith thinks he is able to perform this trick, and he also claims to be able to distinguish his view from that of “another constitutivist”, namely, Christine Korsgaard’s (again, with reference to her 1996 text). According to him, Korsgaard commits “a grave mistake” when she identifies reasons “as the demands to which we are subject in virtue of our necessary identity”. (2015: 193). Instead, we should regard reasons for action as connected with our function as agents. If we, with Korsgaard (as interpreted by Smith), think that reasons for action are those demands to which we are subject insofar as we are the kind of thing that we are essentially (that is, insofar as we share in the respective necessary identity), we will end up with deliberative dilemmas. (2015: 194) For instance, to use Smith’s example, we may think of ourselves as essentially biological human beings facing the demands of reproduction. At the same time, however, we may also see ourselves as agents who have good reasons to help, and not interfere with, each other. The demands of reproduction, on the one hand, and, on the other, those of helpfulness and non-interference may be in tension or even irreconcilable conflict, in some circumstances.

Consider a particular person; as a human being, she will be a biological being necessarily, since, by definition, this identity is essential for human beings. Since the demand of reproduction is part of this identity, the particular person under consideration, as a human being, is, as a matter of fact, subject to this demand. Yet, according to Smith, this demand may come in contradiction with other demands, for which we may see ourselves as having good reasons. Hence, the starting point for a constitutivist account should not be some essential identity, but an account of ourselves as agents, since it is within the framework of such an account of agency that we can talk about commitments and our reasons for these commitments. Hence, according to Smith, the conflicting demands, which seem to be irreducible from the perspective of the presupposition of an essential identity, disappear when the starting point is an account of ourselves as agents and our reasons for commitment. For him, “only the demand relative to our function as agents is analytically tied to the concept of a reason for action”. (2015: 19)

This suggests that there are at least two significant aspects of constitutivism, which must be considered. First, with which account of ourselves the constitutivist begins seems crucially important – on Smith’s account, Korsgaard starts with an account of our essential identity, that is, an account of that without which we would be a different kind of being; yet, being essentially this or that kind of being, with this or that kind of demands upon us (say, biological human beings facing the demands of reproduction) leaves open the question whether being in this way is normatively permissible and desirable.

Secondly, how this account is connected with the normative claims the constitutivist derives seems equally significant, and here Smith specifies that the connection happens “analytically”. As we will see, Korsgaard does not discuss this second important aspect of a constitutivist account and, at least if we interpret an ‘analytic’ connection in the standard way, Smith’s specification of the link between the account of ourselves as agents and the normative conclusions identified as constitutive of action cannot pull any normative rabbit out of the constitutivist hat.

My paper focuses on this aspect of constitutivism, precisely because it is a significant, and yet not much examined, issue. Moreover, as already mentioned, I focus on Korsgaard’s constitutivism, because, although she does not explicitly discuss the status of the connection between the initial account of agency, from which the constitutivist starts, and the later substantial normative account, with which he hopes to conclude, she does make a promising suggestion, which is worth pursuing. Nevertheless, to begin with, let us focus on the first important aspect of constitutivism and examine Smith’s objection to Korsgaard. If this starting point is flawed in Korsgaard, then there is not much interest in exploring further the way in which a substantial normative account could be derived on this basis. I, however, think that Korsgaard’s view on this is not flawed in the way Smith claims.

As mentioned above, on Smith’s account, the problem with Korsgaard’s constitutivism is that it is supposed to begin with an account of agency as given by our essential identity, by the kind of thing we essentially are. The problem is that demands stemming from this essential identity will be in tension or strong conflict with demands provided by reasons we have, as agents, to act in one particular way or another. Yet, as we will see in more detail in Section 4, Korsgaard’s starting point is not an account of our essential identity, which would include some demands to which we would be subject as a matter of fact; Korsgaard agrees that our various identities and roles may provide various demands on us as a matter of fact, but she also regards us as beings that have the capacity to reflect on these demands, to distance ourselves from them and to question them. She thinks that, as self-reflective beings, we are beings that need reasons to act.

This characterisation of human beings as self-reflective beings that need reasons to act asserts the connection between agency and reasons. Hence, if there is some essential identity, which we must necessarily adopt and which forms the starting point for Korsgaard, then this is only an identity as beings who need reasons, since we can question any putative essential identity and try to determine whether to commit to its demand or not. Hence, Korsgaard begins from an account of human beings, which makes it possible for us to question our identities and roles and to justify our commitments to them.

In conclusion, Korsgaard’s view, although admittedly dominated by an understanding of reasons in terms of identity, does not link reasons to some essential identity, which would make us subject to particular demands as a matter of fact. As human beings, as self-reflective beings who need reasons to act, we have an identity, but this identity does not impose specific demands – it questions them by reflecting on our commitments, by evaluating them and by endorsing them, if we have reasons to endorse them. According to Korsgaard:

Circumstances may cause you to call the practical importance of an identity into question: falling in love with a Montague may make you think that being a Capulet does not matter after all. […] What is not contingent is that you must be governed by *some* conception of your practical identity. For unless you are committed to some of your practical identity, you will lose your grip on yourself as having any reason to do one thing rather than another – and with it, your grip on yourself as having any reason to live and act at all. But *this* reason for conforming to your particular practical identities is not a reason that *springs from* one of those particular practical identities. It is a reason that springs from your humanity itself, from your identity simply as *a human being*, a reflective animal who needs reasons to act and to live. And so it is a reason you have only if you treat your humanity as a practical, normative, form of identity, that is, if you value yourself as a human being. But to value yourself as a human being is to have moral identity. (1996: 120–1)

There is here a clear distinction between a person’s particular practical identities (such as, being in love with a Montague) and a person’s identity as a human being (a reflective animal who needs reasons to act and to live). This distinction already suggests that Korsgaard begins with an account of ourselves as agents, as human beings who need reasons for our commitments, but this is not an account, which would regard us as subject to certain demands and reasons for action. On the contrary, as mentioned before, this account makes possible the process of questioning any specific identities and commitments. Moreover, our identity as human beings can itself become the object of reflection and commitment. It is only when we acknowledge that our identity as human beings (as a reflective animal who needs reasons to act and to live, and, hence, as an agent) is valuable that we acquire a moral identity. This moral identity, however, only makes sense from the perspective of reflection and, hence, from the perspective of our humanity.

Hence, our humanity is a capacity, which is not always acknowledged as valuable; we may continue our lives without acknowledging the value of reflection. When we treat this human identity as an identity we should be committed to, we acquire moral identity. But this moral identity is not an essential identity either, since not valuing your human identity is still compatible with your capacity for reflection and agency, and it is compatible with your being an agent, a human being. This, I think, answers Smith’s objection to Korsgaard or at least makes this objection less urgent. Thus, to sum up, our identity as human beings, makes it possible for us to question any putatively essential identity, whereas our moral identity is acquired when we do not simply acknowledge our reflective capacity and the need for reasons, but when we value them.

To be sure, there are here several questions, which are left open, such as: In what sense does Korsgaard claim that we “need” reasons to act and to live, given that she also acknowledges that we may not value our reflective capacity and agency? Is it really the case that our moral identity is not an essential identity – after all, if we needed our humanity, then should we not value it and, if so, then would moral identity not be a necessary part of our identity as agents? We will see that such questions will emerge in one form or another in the reconstruction and evaluation of Korsgaard’s argument for normativity in Sections 4 and 5; in the next section, however, I turn to the second important aspect of constitutivism mentioned above: the connection between, on the one hand, the account of agency, which is the starting point for a constitutivist, and, on the other, the normative claims she thinks she is able to derive from that account of agency.

## The Constitutivist Trick

Smith claims that the concept of an agent’s reason for action “is not basic”, but “gets analysed” in terms of the concept of desirability-relative-to-that-agent. The latter, in its turn, is not basic, but gets analysed in terms of the desires of that agent’s idealised counterpart. Constitutivism enters the picture, Smith argues, when we ask ourselves whether the concept of ideality (the desires of the agent’s idealised counterpart) is basic. He thinks the notion of the agent’s idealised counterpart should in fact be that of an agent’s functioning optimally as a desire-realiser. (Smith 2015: 189) Smith’s version of constitutivism claims to be able to derive several conclusions from this notion of functioning optimally as a desire-realiser, some of them representing substantial reasons for action.

For instance, from an understanding of agents as desire-realisers, we can allegedly deduce the fact that agents who perform their function optimally must have and exercise the capacity to realise their desires, no matter what their content, and know the world, no matter what the world is like. From this, Smith claims, it follows that agents must possess coherence-inducing desires to help and not interfere, if agents are to perform their function optimally. We are also told there is an “analytic tie between facts about what is desirable-relative-to-an-agent and facts about the desires of ideal agents”, as well as between “facts about an agent’s reasons for action, on the one hand, and facts about which options that agent has and what is desirable-relative-to-that-agent, on the other”. (Smith 2015: 193)

Smith offers a complex argument, but, for the purpose in my paper, the important point is the link between an account of an agent as desire-realiser and the substantial normative claims derived as constitutive of agency: how do we get from an account of agency to substantial normative conclusions? As we have seen, there are several steps for the derivation of the substantial normative claims made by Smith, and some are presented explicitly as made analytically. It is not clear what Smith has in mind when he talks about an implication’s being drawn analytically, but the following standard account might clarify this notion sufficiently for the purpose of this paper.

The distinction between analytic and synthetic connections has a relatively long history. My attempt here is to work with a minimal conception of this distinction, which would be as uncontroversial as possible. According to Kant:

In all judgements in which we think the relation of a subject to the predicate (I here consider affirmative judgements only, because the application to negative judgements is easy afterwards), this relation is possible in two ways. Either the predicate B belongs to the subject A as something that is (covertly) contained in the concept A; or B, though connected with concept A, lies quite outside it. In the first case I call the judgement *analytic*; in the second, *synthetic*. Hence (affirmative) analytic judgements are those in which the predicate’s connection with the subject is thought by [thinking] identity, whereas those judgements in which this connection is thought without [thinking] identity are to be called synthetic. (A6-7/B10)^7^

I am going to assume that we can regard claims in general as consisting of a subject and a predicate – the subject indicates that to which the claim attributes the predicate. For instance, the claim that ‘desire-realising agents who perform their function optimally must have and exercise the capacity to realise their desires no matter what their content and know the world no matter what the world is like’ can be seen as attributing a predicate (having and exercising the capacity to realise desires no matter what their content and knowing the world no matter what the world is like) to the subject (the desire-realising agents who perform their function optimally).

This subject itself can be understood as a claim (‘desire-realising agents perform their function optimally’), which attributes a predicate (performing their function optimally) to the subject (desire-realising agents).^8^ One example of an analytic judgement Kant offers is that all bodies are extended – we think the predicate of being extended as contained in the concept of a body. By contrast, the claim that all bodies are heavy does not make explicit a predicate (about the heaviness of a body) that we think as already contained in the subject (the notion of a body); hence, the claim must connect subject and predicate by linking them through a further element. This element can, in some cases, be given by experience. For instance, for ‘This book is red’, the truth of the claim depends on an experience of this book as red.

Hence, Kant claims, in the case of analytic judgements, the predicate is not different from the subject, in the sense that it is included in the subject. This is why Kant says that affirmative analytic judgements are those in which the predicate’s connection with the subject is thought by thinking identity. By contrast, in the case of synthetic judgements, the claim attributes to the subject something that does not already exist in the notion of the subject. One implication of this is that an analytic judgement would not be able to make a substantial claim, a claim asserting about the subject something beyond what is already asserted by the subject. By contrast, a synthetic judgement will assert something beyond what the subject already includes.^9^

An analytic claim or judgement is a claim which clarifies the concept of the subject by making explicit at least a part of it – it does not provide anything that is not already included in the concept of the subject (although, of course, a particular person may not be aware of the predicate as part of the subject and learns something new through the analytic judgement). By contrast, a synthetic judgement will assert something new – it will connect the concept of the subject with another concept, which is not already included in the subject.

One implication of this is that analytic judgements have a necessity, which is not to be found in synthetic judgements. To deny an analytic judgement is to deny that part of the concept of the subject (the predicate) is part of the concept of the subject, which is contradictory and, hence, impossible. If to deny an analytic judgement is to commit to impossibility, then the analytic judgement is necessary and, hence, Kant says, a priori. By contrast, synthetic judgements do not assert a predicate already included in the subject, so denying that the predicate applies to the subject does not commit us to an impossibility. Given the contingency of experience, analytic judgements cannot be based on experience, whereas synthetic judgements can:

Thus the [analytic] proposition that bodies are extended is one that holds a priori and is not an experiential judgement. For before I turn to experience, I already have in the concept [of body] all the conditions required for my judgement. I have only to extract from it, in accordance with the principle of contradiction, the predicate [of extension]; in doing so, I can at the same time become conscious of the judgement’s necessity, of which experience would not even inform me. (B11-12)

One of the crucial aspects of Kant’s distinction is that, although he thinks all experiential judgements are synthetic, he does not think all synthetic judgements need to be experiential, that is, based on experience or a posteriori. The idea of a synthetic a priori judgement is what gives Kant hope that substantial judgements can be necessary and, hence, that cognition is possible. Insofar as we take cognition to refer to those claims, which are substantial and necessary, they can only be so if they are synthetic and a priori. Kant thinks the propositions of mathematics are synthetic and a priori. For Kant, a claim such as ‘7 + 5 = 12’, for instance, is synthetic, although it is also a priori.^10^

Kant’s account of the distinction between synthetic and analytic propositions has been criticized in various ways. For the purpose of this paper, I cannot review the debates and defend any particular aspect. What I hope to do is to focus on a sufficiently unproblematic version of the distinction, while assuming that the distinction can still be used.^11^ The first element of this simplified account is an assumption that declarative and imperatival propositions (whether affirmative or negative) can be understood as having two parts: a subject and a predicate. Secondly, there is a claim that, in the case of the analytic propositions, the link between subject and predicate is of identity.^12^ If the distinction between analytic and synthetic propositions is to be exhaustive, then synthetic propositions would be those where the relation between subject and predicate is not one of identity.

One implication of this basic account of the distinction is already familiar: analytic propositions are not substantial claims: by linking subject and predicate, they do not assert anything more than what is already presupposed by the subject of the propositions. A second, already mentioned implication is that analytic propositions are necessary: their negation is a negation of a relation of identity and leads to a contradiction. As we know from standard modal logic, it is the negation of necessity that leads to impossibility, so analytic propositions are necessary, given that their negations lead to contradictions. Finally, given their necessity, analytic propositions are a priori, since a posteriori, experiential propositions are contingent and, hence, cannot be necessary.

With this background in place, the worry of an anti-constitutivist, like Wiland, can be expressed more specifically in the following way. The issue is one of performing magic, because, if the premises from which the constitutivist starts already include the substantial account of normative reasons for action that the constitutivist claims to be able to derive (for instance, the desires to help and not interfere mentioned by Smith), then these premises are not really abstract, although as we have seen in the definition of constitutivism, they should be so. If those premises do not already include this substantial account, then a substantial account cannot be derived from them analytically. If it is not derived from them analytically, then it should be derived synthetically and, yet, if it is to be derived synthetically, then the validity of the derivation will be either contingent (for instance, when based on experience) or necessary (in which case it becomes a synthetic a priori derivation).

What is specific for a substantial *normative* account, however, is its necessity: the claim that something *should* be the case (whether ethically, aesthetically or from some normative perspective) is stronger than the claim that that thing is the case and implies a specific requirement. Yet, in Smith’s account, it is unclear how such a derivation is to be obtained and no indication that one of the premises in the argument would be synthetic a priori.

Smith does not examine the nature of the constitutivist argument, which is supposed to take us from some abstract premises about agency to some substantial normative conclusions, although he does present the argument in detail; nor does Korsgaard examine the nature of the constitutivist argument, although she does indicate a promising avenue, which I will investigate in the next three sections. This is the promise of a transcendental argument, which would take us from abstract premises to substantial normative claims. Transcendental arguments have been discussed in the literature and they are still a topic at issue. There is yet no overall agreement with regard to their role, their structure and how they are supposed to function. They are, therefore, perhaps most appropriate for the task of performing the magic expected from constitutivism.

## Korsgaard’s “Transcendental” Argument

Consider the following discussion of a transcendental argument offered by Christine Korsgaard in *The Sources of Normativity* (1996). First, according to her, we can assume that the human mind is “essentially self-reflective” (92). This means that we have a capacity to turn our attention to our own mental activities, “to distance ourselves from them and to call them into question”.^13^ (93) This threefold process of self-reflection, distancing and questioning enables us to have control over our actions: we can act only by motivational factors, which we decide to endorse or which we decide not to oppose. We can in this way control our incentives and, generally, any other factors that may prompt us to act. In order for such mental activities to be motivational, that is, in order for them to motivate us, we need to endorse them (or at least indirectly to endorse them by allowing them) to be determining grounds of our actions.

For instance, I may have a powerful desire to play computer games at the moment; upon reflection, distancing and questioning, I realise that, given an already made commitment to finishing this article by a certain deadline, I should continue to work. Hence, a mental activity, like my desire to play, will motivate me to act (and leave my work), if I endorse it or at least if I do not oppose it. The process whereby I decide whether to allow this desire to be a ground for my leaving my work for play may involve reflection on that desire, as well as reflection on other reasons I might have for or against it.^14^

Secondly, let us suppose I act; this happens if the questioning reflective process ends with an answer. I may conclude the questioning process with the outcome that I should act on one of the motivational mental activities (that particular desire or some other inclinations or predispositions). Given that I could also have acted on other motivational mental activities, the fact that I acted in this way and not in some other way indicates that I had a reason to endorse this motivation (even when this reason was that of *illustrating* an action which is performed in a particular way for *no* specific reason).

Hence, the conclusion of the reflective process is meant to provide me with a normative result: the obligation, permission or prohibition, which I have reason to endorse or to allow, is right.^15^ For instance, I now have a reason to continue to work, rather than play computer games, *because* I have a previous commitment to completing my work by a particular deadline *and* leaving my work to play would make the completion of the project by the specific deadline less likely. My reason provides a justification, which usually makes reference to a principle or value (carrying out what one committed oneself to do is good) and indicates what seems to me to be what I should do. ‘Reason’, therefore, is a “normative word “, which “refers to a kind of reflective success”. (93) The same, Korsgaard says, goes for the words “good” and “bad”. (94)

Now, if reasons are the result of reflective success, the next question is how I can lead the reflective process to a successful conclusion. Given that the process of reflective questioning is one, through which I choose which motivational element to act on, the answer, according to Korsgaard, is that “the principle or law by which you determine your actions is one that you regard as being expressive of *yourself*”. (100) For instance, in deciding that I should complete what I committed myself to completing, I assume that the principle of keeping promises is valuable for me. This, in its turn, means that the principle functions as *my* principle of action, and, in this sense, it is expressive of me.

That this principle is expressive of me is another way of saying that I take it to be justified, that it is right to act in this way given the kind of person I am and the situation where I find myself. As an implication, we can say that I act based on an evaluative identity or, in other words, based on a view of myself which I value. It is under such a view that, according to Korsgaard, I find my life “to be worth living” and my actions, “worth undertaking”. (101)

One extreme illustration of this account that Korsgaard offers is a reaction against a “threat of a loss of identity”. (102) A loss of identity seems to be behind claims such as: “*I* cannot do this”, when an action that seems morally abhorrent is suggested to a moral agent. For the agent feels that acting in that way would mean abandoning some fundamental principle, a principle without which she would not be the same person and without which perhaps her life would not be worth living. Hence, a person may be capable of sacrificing herself, when the alternative would be conceived as incompatible with the kind of person she is.^16^ We should bear in mind, however, that, in many cases, our reasons for action derive from contingent and local identities. We belong to various groups and clubs, and our memberships change periodically, sometimes without any thoroughly considered reasons and sometimes even when, as in the extreme case presented above, they seemed to be aspects of our identities without which we could not exist.^17^

Thirdly, and as a result, one way to understand scepticism, Korsgaard says, is as “the fear that we might not find what Kant called ‘the unconditioned’.” (94) Hence, one problem is to account for normative claims, which are not conditional on local and contingent identities. To account for such a claim, we can start with some contingent identities: we are born in a certain family or community, and sometimes into a profession or craft, we form ties to other persons, movements and ideas, and, as contingent, many of these identities can be shed. (120) Yet, if all these identities can be shed and no identity has an unconditional character, then the sceptic may question whether there is anything more than contingent standards in morality.

In answer to this, Korsgaard suggests that what is *not* contingent is that an agent must be governed by *some* conception of practical identity, by *some* project. Without an identity, even a local and contingent one, there is no reason to do one thing, rather than another one and, hence, no reason to act at all. Yet, the commitment, or rather meta-commitment, to having *some* practical identity is not derived from one of the contingent identities: “it is a reason that springs from your humanity itself, from your identity simply as *a human beings*, a reflective animal who needs reasons to act and to live”. (121)

On Korsgaard’s account, therefore, in order to answer the sceptical worry we need to account for an unconditional requirement (whether an obligation, permission or prohibition^18^). Because such a requirement stems from reasons, which are provided by practical identities, the unconditional obligation will have to stem from a practical identity, which is necessary. We have seen that, as self-reflective beings, having *some* practical identity is necessary, if we are to be able to act. Hence, as agents, we are committed to the non-contingent requirement of having some practical identity. This non-contingent requirement is part of our identity as human beings; hence, it is part of our humanity. Identities which provide reasons (whether conditional or not) are evaluative, and, in order for them to be evaluative, we need to be committed to their validity. Given that the non-contingent requirement, to the validity of which we must be committed, is part of our humanity, it follows that we must be committed to our humanity or, in other words, “humanity […] must be valued”. Given that this is a non-contingent identity, it “must be valued for its own sake”. (122)^19^

This, according to Korsgaard, can answer the sceptic: if the sceptic is a human being and if he agrees with her argument, then the sceptic must value his humanity, if he is to act at all:

Since you are human, you *must* take something to be normative, that is, some conception of practical identity must be normative for you. If you had no normative conception of your identity, you could have no reasons for action, and because your consciousness is reflective, you could then not act at all. […] The argument I have just given is a transcendental argument. (123)

Korsgaard’s transcendental argument can therefore be summarised as follows: the sceptic doubts the possibility of an unconditional requirement – he doubts (what we can call) practical normativity; yet, human beings are reflective beings, who need reasons to act^20^; reasons to act are provided by evaluative identities (identities we value) and, as evaluative identities, these identities are particular instantiations of the unconditional requirement that, as human beings, we be committed to some identity. Yet, as a human being, the sceptic must himself act and, in order to do so, must rely on some unconditional evaluation – her identity as a human being; but this contradicts the sceptic’s initial doubt, which must therefore be rejected.

If this is a transcendental argument, as Korsgaard claims it would be, then how exactly is it supposed to work?

## A Reconstruction of Korsgaard’s Argument

Consider a standard account of explorative or deductive transcendental arguments:

1. X.
2. Y is a necessary condition for the possibility of X.
3. Therefore Y.^21^

What are X and Y in Korsgaard’s argument? She relies on the fact that human beings act and that a necessary condition, which makes possible actions for human beings, is that they be committed to some evaluative identity. Yet, this commitment to some evaluative identity is unconditional. Hence, if the sceptic is an agent (if she acts), then she cannot deny practical normativity, that is, she cannot deny the unconditional requirement provided by such an evaluative identity.^22^

Korsgaard’s argument can therefore be understood as a deductive transcendental argument in the following way (call this CA_1_ or the Constitutivist Argument_1_):^23^

- P_1_. Human beings act.
- P_2_. A necessary condition for the possibility of a human being’s action is a commitment to an unconditional principle or value (a standard).
- C. Human beings are committed to an unconditional standard.

This interpretation seems to be confirmed by Korsgaard’s reformulation of the transcendental argument:

I might bring that out more clearly by putting it this way: rational action exists, so we know it is possible. How is it possible? And then by the course of reflections in which we have just engaged, I show you that rational action is possible if and only if human beings find their own humanity to be valuable. But rational action is possible, and we are the human beings in question. Therefore we find ourselves to be valuable. (Korsgaard 1996: 123–4)^24^

So Korsgaard does start from our actions as human beings, then identifies the necessary condition which makes action possible and then asserts this necessary condition. Since this necessary condition is that of being committed to an unconditional standard of action, and since this unconditional standard is part of our evaluative identity as human beings, it follows that we are committed to our humanity. The sceptic, insofar as she is an agent (that is, insofar as she acts), cannot doubt that she is committed to an unconditional standard and, hence, cannot doubt that there is normativity.

Now, consider the first premise in CA_1_: Human beings act. This is a factual statement and usually the truth of factual statements is contingent. Necessary conditions depend on the truths for which they are conditions, so the necessary condition of acting will depend on the contingent truth of acting. This also implies that the (necessary) condition, which makes possible the contingent truth, will be a contingent truth, affected by the contingency of that for which it is a condition. Specifically, the commitment to the unconditional standard of humanity will be made manifest when we act, and if acting is contingent, being committed to the unconditional standard of humanity will also be contingent. This leaves it open for the sceptic to say that, although acting in the way in which Korsgaard understands it may presuppose a commitment to an unconditional standard, there seems to be no compelling reason to perform actions in that way.

The contingent character of the standards associated with my actions is one of the important issues in the literature on Pyrrhonian scepticism. For, according to the Pyrrhonian sceptic, it is possible to act without being committed to a particular belief, let alone to an unconditional standard: “Holding to the appearances, thus, we live without beliefs but in accord with the ordinary regimen of life, since we cannot be wholly inactive”. (Sextus Empiricus 1996: 92) Moreover, the model of agency that this form of scepticism advances is significantly different from that suggested by Korsgaard; the Pyrrhonian sceptic does not seem to be prompted by some evaluative identity, but by a form of assent that, while considering appearances, neither asserts nor denies them: “…for the sceptic does give assent to the *pathé* that are forced upon him by a *phantasia*; for example, when feeling hot (or cold) he would not say ‘I seem not to be hot (or cold)’“. (Sextus Empiricus 1985: 90)

The extent to which this alternative conception of action is viable is a topic at issue, and it would take me too far away from the topic of this paper to try to explore it here in any depth.^25^ The important point to be drawn from this very sketchy discussion of scepticism is that, as long as the claim of a commitment to an unconditional standard is premised on a contingent truth, the sceptic has the legitimate option to doubt normativity (where ‘legitimate’ simply means non-self-undermining), since by regarding action as contingent he can also regard any commitment to an unconditional standard as contingent too.^26^

But let us assume that action in the first premise of CA_1_ is not contingent: Human beings act, but *necessarily* – for instance, agency is a necessary condition of humanity, so that, human beings can only act.^27^ As Korsgaard puts it in a more recent text, in a formulation with a distinctively existential flavour: “Human beings are *condemned* to choice and action”. (2009: 1 – emphasis in the original)^28^ If we understand the first premise of CA_1_ in this way, namely, if we take it to involve a claim to necessity, then the transcendental argument with which we started becomes:

- P_1_’. Human beings necessarily act.
- P_2_. A necessary condition for the possibility of a human being’s action is a commitment to an unconditional principle or value (a standard).
- C’. Human beings are necessarily committed to an unconditional standard.

Let us call this CA_2_. Compared to CA_1_, CA_2_ only introduces a claim of necessity in premise P_1_’, a claim which, given that CA_2_ is valid, is preserved in the conclusion C’.

One worry about this argument is that the first premise specifies action as a necessary feature of humanity; this is worrying since action may be right, wrong or morally indifferent. Assuming we understand ‘human being’ in a classificatory, rather than evaluative, sense, we will say that a human being can perform any of these three types of action. In this case, CA_2_ claims that, in order to perform an action (whether right, wrong or morally indifferent), a human being must be committed to an unconditional standard. It follows as a result, however, that this standard will not have a specific content – it is a necessary condition of action, irrespective of whether the action is right, wrong or morally indifferent. Yet, then, even if the argument is correct, it cannot challenge the sceptic’s claim about *normativity*.^29^ Moreover, if the aim is not simply that of proving the sceptic wrong, but also that of providing guidance for moral action, then this type of argument has no chances to succeed, since what it tries to establish are necessary conditions of action, including wrong actions, rather than action-guiding standards.

This suggests the following solution: if the first premise is understood as a statement specifically about right actions, then there is a chance for an argument for normativity and for specific guidance. I have mentioned above that in some formulations of her transcendental argument, Korsgaard refers not only to action, but to rational action. This, I think, is precisely what can motivate this change from a talk about actions to a talk about rational actions: if we take seriously Kant’s claim that an action performed under the guidance of pure practical reason or the will is a moral action, then to talk about rational action is to talk about moral action too.^30^ In this case, the transcendental argument becomes:

- P_1_”. Human beings necessarily perform rational actions.
- P_2_’. A necessary condition for the possibility of a human being’s performing rational actions is a commitment to an unconditional standard.
- C’. Human beings are necessarily committed to an unconditional standard.

I make haste to note that, compared to P_2_, P_2_’ is unproblematic – it is just P2 restricted in scope from reference to actions in general to an application to rational actions only. P2’ may not be interesting, since it is weaker than P2, but, in the context of the discussion of P2, it is unproblematic as far as its content is concerned. Thus, since a necessary condition for performing actions in general is a commitment to an unconditional standard, a necessary condition for performing right actions will also be a commitment to that unconditional standard. I will call this version of the argument CA_3_.

CA_3_ seems able to avoid the problems of CA_1_ and CA_2_: it asserts a commitment to an unconditional standard as a necessity, and it refers to rational or right actions and their necessary condition. The problem with CA_3_ emerges when we pay closer attention to the response this argument offers in fact to the sceptic. For this response does not seem any more able to answer the sceptic and to offer practical guidance than the response offered by CA_2_ does. For recall that the unconditional standard that is the necessary condition for the performance of right/rational actions is that of following *some* principle or value. This, however, is not informative, since it seems *any* principle or value would do.

To be sure, the claim made by the unconditional standard is not that it is *sufficient* to follow a wrong principle in order to perform a rational action – this would be counterintuitive; the claim is only that the performance of a rational action presupposes as a *necessary* condition the commitment to some standard – and this does not exclude wrong principles and evil values. Hence, although the conclusion might not be counterintuitive, it is not substantial either.

Moreover, as in the case of CA_2_, we might try to argue that, although CA_3_ does not conclude with our commitment to a substantial standard, nevertheless it does conclude, against the sceptic, with our commitment to an unconditional (even if only formal) standard. Yet, once CA_3_ is restricted in its first premise to rational or right actions, it can no longer function as an answer to the initial sceptical challenge concerning unconditional normativity. For the argument seems to presuppose that there is something called right action that human beings necessarily perform, whereas the sceptic doubts unconditional normativity in general. This seems to suggest a serious problem for any attempt to construct such a transcendental argument: if the aim is to show that there is normativity, then the sceptic cannot accept that we necessarily perform right actions, so the starting point must be a necessity of acting in a more general sense, not restricted to the normative domain. In this case, however, if the transcendental argument is supposed to show that the sceptic herself must be committed to an unconditional standard, then this implies that we must accept the possibility of deriving an ‘ought’ from an ‘is’. I take this to be problematic.^31^

Be that as it may, I would like to argue that constitutivist arguments may still be able to provide results, which are action-guiding, if the necessary condition for the performance of rational actions is formulated as a more substantial moral standard. In fact, this is what Korsgaard eventually plans to show, namely, that we should follow the Categorical Imperative^32^, as a necessary condition for the performance of (rational) actions. (98–100)^33^ Let us assume this new move succeeds and she can show that a commitment to the Categorical Imperative is the necessary condition which makes possible rational actions. The constitutivist argument, which is the focus of this paper, becomes:

- P_1_”. Human beings necessarily perform rational actions.
- P_2_”. A necessary condition for the possibility of a human being’s performing rational actions is a commitment to the Categorical Imperative.
- C”. Human beings are necessarily committed to the Categorical Imperative.

Let us call this CA_4_; this seems to be the best version of the transcendental argument offered by Korsgaard, or at least a version which is better than CA_1–3_. There is of course a longstanding debate on the extent to which the Categorical Imperative is in any sense a substantial or informative standard. As I have mentioned, there is also a question concerning the extent to which Korsgaard can successfully show that the Categorical Imperative is indeed a necessary condition for the possibility of morally right actions. I am going to set aside these important issues, in order to focus on the main topic of this paper: assuming the Categorical Imperative is action-guiding and assuming the sceptic does not doubt normativity per se, but the ability we have to formulate specific standards which have justification, does the constitutivist argument offer an answer to the sceptic?

## An Evaluation of Korsgaard’s Argument

First, I am going to assume that P_2_” is correct^34^, in order to focus on P_1_”. I am focusing on this premise, since it seems to make a very strong claim. It is unclear in what sense we can say that we necessarily perform rational actions. Any case of weakness of will or evil action shows the contrary. We could understand the claim as more limited – say, human beings necessarily perform *some* rational actions. But this interpretation would defeat the argument’s purpose: we are interested in refuting the sceptic and getting practical guidance; if the conclusion of the argument will be that human beings are necessarily committed to the Categorical Imperative in some instances, then this would no longer be a commitment to an unconditional standard. If the validity of a standard were limited to certain instances, then the standard would no longer be unconditional.

To be sure, there are instances where we may not be under the obligation of acting under the Categorical Imperative – say, when we are asleep.^35^ But this is because when we are asleep we are not treated as responsible agents. This may suggest a better interpretation of P_1_”: Human beings necessarily perform rational actions, insofar as they are human beings. Although this may account for situations where we may be justified in not treating one person as a responsible agent, it is still not enough to make the premise plausible. This is because even in cases where we can legitimately treat a person as morally responsible, it may still be possible for that person to act irrationally or against reason. Only if we take’human being’ to mean not simply a responsible being, but a responsible rational or morally good human being, can P_1_” be more palatable.

Yet, on this interpretation of P_1_”, where ‘human being’ is taken in an evaluative sense, the claim is almost tautological: namely, the claim is that human beings, in the sense of responsible and rational, good beings, necessarily perform rational actions. It is unclear to me how, on this reading, the argument will help in any way with a response to the sceptic’s challenge concerning unconditional normativity, since this seems to be already assumed. Nevertheless, the argument might provide a response to the question of moral guidance – it might be that the conclusion could provide an unconditional standard to which human beings, in the evaluative sense of the expression, must be committed. For recall that the two issues considered here in relation to the skeptical challenge is the commitment to an unconditional standard and the possibility of specifying the standard or standards to which we should be so committed – so while the argument cannot respond to the former issue, it might still be able to respond to the latter.

Let us now move on to P_2_”: A necessary condition for the possibility of a human being’s performing rational actions is a commitment to the Categorical Imperative. As already said, I am going to assume this is correct; what I am interested in at this stage is the way in which it is correct. In particular, I am interested in the nature of the necessity involved in this claim. What does it mean to say that the commitment to the Categorical Imperative is a *necessary* condition which makes possible a human being’s performing rational actions? Let us return to the analytic/synthetic distinction. P_2_” is a judgement expressing a necessity and, as we have seen, necessities can be formulated in two ways^36^: either in the form of an analytic judgement or in the form of a synthetic a priori judgment.

Most recent Kantian philosophers have tried to provide accounts of some of Kant’s insights, but without the metaphysics of transcendental idealism. A synthetic a priori judgement, on a standard interpretation, needs the Kantian metaphysics; insofar as such a judgement is synthetic, it can only be made a priori if the two parts of the judgement which are connected synthetically are connected either through an a priori intuition or through the idea of freedom. For instance, according to Kant, ‘7 + 5 = 12’ is synthetic a priori because it is a substantial claim. As we have seen^37^, we cannot derive the notion of twelve from the notion of the addition of the numbers 7 and 5. We get to this result by constructing through our a priori intuitions (either through a spatial or through a temporal representation, but in the case of this example, through a temporal representation of the numbers and their sum) the mathematical operation and its result.

Whether we regard an a priori intuition or an idea of reason as able to connect synthetically and a priori the parts of the judgement, we would need to accept at least parts of Kant’s metaphysics. This is because space and time (as a priori intuitions) and freedom (as an idea of reason) could unify parts of a judgement only if we regarded them as structural elements of the mind, and this, I would argue, presupposes at least some important parts of transcendental idealism.^38^

If so, then one alternative is to regard P_2_” as analytic and to understand the necessity of the commitment to the Categorical Imperative as the result of the fact that this commitment is already presupposed by the notion of performing rational or moral actions. In the same way in which a necessary condition for the possibility of being a bachelor is to be unmarried, a necessary condition for the possibility of performing rational actions is to be committed to the Categorical Imperative. To make this more palatable, we can rephrase the condition so that it is not expressed directly as a Categorical Imperative, but as, say, a principle of universalisability or of not treating others merely as ends for our purposes.

But this has problematic implications for CA_4_. Thus, if part of being human (in the evaluative sense) is to perform rational actions and if performing rational actions means being committed to the Categorical Imperative, then CA_4_ only makes explicit something that was already implicit in the evaluative sense of the notion of a human being. One possibility is for the sceptic to agree with this notion of a human being, but, then, she does not need an argument to accept a commitment to an unconditional standard. The other possibility is when she does not agree with the evaluative notion of human being, in which case CA_4_ cannot help, since all CA_4_ does is to make explicit something already included in the evaluative notion of human being.

The only alternative left, then, is that P_2_” would represent a claim to necessity provided by a synthetic a priori judgement. For instance, rational agency and the Categorical Imperative could be connected through freedom. Since the Categorical Imperative is a law of freedom and action presupposes freedom, we can perhaps assert P_2_” as a synthetic a priori judgement. As a synthetic judgement, P_2_” would assert something about the rational actions of good human beings, which would not already be included in these notions. If the necessary condition of rational action is a commitment to an unconditional standard, and if this commitment is not simply a commitment to something already presupposed by the notion of a morally good human being, then the argument can refute the sceptic and provide an action-guiding moral criterion. This would then be the path that would need to be followed by a constitutivist in order to offer a substantial account of normative reasons or standards of action and at the same time to refute scepticism about such an account by starting from premises that are sufficiently weak to be acceptable also to the sceptic.

## Conclusion

In this paper, I have argued that constitutivism can represent a genuine avenue for constructivist attempts to formulate normative standards without realist metaphysical presuppositions and in a non-arbitrary, principled way. I have focused specifically on Korsgaard’s constitutivism and on an epistemic aspect of the debate concerning constitutivism. More precisely, I discussed whether it is possible to construct substantial normative standards through an argument with premises, which are less substantial normatively. In general, supporters of constitutivism argue that such a justification of normative standards is possible and they present various arguments as illustrations of how such a justification is to be carried out, yet they do not examine the nature of such an argument. This leaves them open to the accusations that what they are trying to achieve is only possible by magic.

Unlike other supporters of constitutivism, Korsgaard makes an important suggestion about the nature of such an argument – she thinks a transcendental argument can perform the magic of pulling the normative rabbit out of the constitutivist’s hat. If successful, such an argument would enable her to answer the sceptic who doubts the existence of unconditional practical standards. It would enable her to answer also the related question of the possibility of deriving robust practical norms from slender premises, which represents a version of the classical problem of empty formalism any Kantian moral theorist needs to face.

I began with an objection to Korsgaard’s constitutivism in *The Sources of Normativity*, an objection according to which, in that text, Korsgaard’s starting point is an account of persons as essentially defined in a way which attributes them, as a matter of fact, some demand. This is in fact a quarrel in the family, as it is a supporter of constitutivism that formulates it. It is, therefore, not so much an objection to the promise of constitutivism, as to the particular version formulated by Korsgaard. However, we have seen that this criticism is unwarranted and very likely generated by Korsgaard’s talk of reasons as deriving from specific identities and roles we are committed to in our everyday life. The criticism is unwarranted, because, although our identities and roles do come with particular demands, the source of our commitment to them is grounded in a more fundamental identity we have as reflective beings, who need reasons in order to commit to particular roles and projects, and in order to act. Korsgaard takes as starting point this more fundamental identity, which sets us the task of justification and reason-providing, rather than attributing to us a particular demand as already justified and normative.

After an excursus aiming to bring more clarity to the conceptual framework, in particular the analytic-synthetic distinction, I have presented in more detail Korsgaard’s account of agency, I reconstructed her transcendental argument and I have evaluated the strongest version. The evaluation of the argument has shown that the crucial aspect of the argument is the premise, which links the starting point (the account of agency) and the conclusion (the commitment to the normative standard). This premise needs to be synthetic and a priori, if it is to enable the argument to function in the expected way. Yet, in order to be able to justify a synthetic a priori judgement, we need to adopt some form of transcendental idealism.^39^

Whereas such a commitment would still be compatible with the constructivist’s intention to avoid metaphysical realism in practical philosophy and arbitrary decisions about the normative standard to be observed, it would go in a different direction than that intended by Korsgaard, who has been careful to avoid commitments to transcendental idealism. Quite independently of this, transcendental idealism has been criticised in various ways; one longstanding and reiterated criticism has been that it fails for offer a distinct metaphysical position and ultimately collapses into, and becomes indistinguishable from, traditional idealism with all its problems. While I think the latter criticism is unwarranted, all this shows is that a viable constitutivism comes with what are standardly regarded as quite high costs.^40^

## Footnotes

1 Thus, “the importance of constructivism […] resides in the insight that the nature of practical truths should be explained in terms of the constitutive features of practical reasoning”. (Bagnoli 2016) Of course, there are various accounts of what constructivism and constitutivism are. This means, however, that on some accounts, certain versions of constitutivism would be incompatible with certain versions of constructivism. For instance, on some accounts, Kant’s version of constitutivism is quite different from Rawls’s version of constructivism. (Krasnoff 1999)

2 Commentators acknowledge the significance of constitutivism irrespective of its contribution to the realism/anti-realism debates, yet the focus continues to be on these debates. (Bagnoli 2016: esp. 7.3) The fundamental objection is that if the construction is not normatively constrained, then norms are arbitrary; if it is normatively constrained, then it relies on some normative constraints which are not constructed (for instance, Shafer-Landau 2003: esp. Ch. 2). Some debates focus on ethical intuitions, as such constraints, and the extent to which they can be taken as normative foundations. (Scanlon 1998: esp. Ch. 5; 2003) Some constructivists do not see a role for ethical intuitions in the construction of norms. (O’Neill 1989; Hare 1983) One way such constructivists respond to the fundamental objection is by claiming that constructed norms are constitutive features of practical reason. (Korsgaard 1996). Korsgaard’s use of the language of transcendental arguments suggests to some that her position is realist. (Crisp 2006: esp. Ch. 2, Sect. 2; Larmore 2008: esp. Ch. 5, Sect. 5; Galvin 2011) The claim that to value ourselves presupposes as a necessary condition valuing humanity is criticised as presupposing that a condition of a thing’s value is itself valuable. (Kerstein 2001; Ridge 2005; Coleman 2006). Whereas constitutivism may distinguish itself from non-naturalist realism (Shafer-Landau 2003) and naturalist realism (Firth 1952; Railton 1986) by regarding norms as the result of a process of construction undertaken from the first-person perspective (O’Neill 1989, Korsgaard 2003, 2009; Street 2010; Velleman 2009), a worry remains that it relies on moral assumptions it does not justify eventually (Raz 2003: esp.; Timmons 2003) One reply here is that the objection misses the point of constitutivism, where the method is circular, but not in a vicious manner. (O’Neill 1992) A further objection is that constitutivism is unsuccessful in deriving norms of morality from features of rational agency;. (Cohen 1996; Bratman 1998; Gibbard 1999; Fitzpatrick 2005; Setiya 2007; Bagnoli 2009). Some such objections are formulated from a realist perspective, whereas others are formulated from an anti-realist standpoint. For the full discussion, see Bagnoli (2016: esp. 7.3).

3 A clarificatory note is in order: I see the constructivist-constitutivist problem of deriving a substantial account of normative reasons from an account of the nature of action and agency as a particular case of the more general problem of deriving substantial conclusions from more abstract and formal premises. Nevertheless, there is a sense in which this second problem does not really make sense. The reason is the following. As we will see in Section 5 of this paper, I present such an argument as a valid syllogism. Yet, a valid logical argument can never derive more substantial conclusions from slender premises. Premises are entailed by the conclusions. The problems makes sense if we regard them in the following way. Consider an argument with the premises A and A- > B; if B is already part of A, then the conclusion B does not add anything to A; by contrast, if B is not already included in A, then the argument shows something substantial. To be sure, the conclusion is already included in the premises, but the point is that in the formulation of the second premise (A - > B), we derive from B something which was not already in A, as opposed to simply making A more explicit.

4 Since the paper focuses mainly on this text by Korsgaard, in what follows, references to this text will be made by indicating the page(s) referred to only.

5 My claim here is that, whereas Korsgaard talks explicitly about a transcendental argument in *Sources*, she abandons the expression in subsequent texts, perhaps also as a result of inviting in this way the criticism that she is getting close to moral realism. See also n2 above. Nevertheless, according to some authors, she continues to use transcendental arguments, even when she is not using this label for them – see n33 below.

6 Quoted in Smith (2015: 187). Smith addresses here Wiland’s and David Enoch’s objections to constitutivism (Enoch 2006), the former’s being formulated against Smith’s version of constitutivism, whereas the latter being broader in focus.

7 In what follows, when referring to Kant’s (1996) *First Critique*, I will do so by using the standard ‘A/B’ (first edition/second edition, in volumes 3 and 4 of the Akademie edition Kant (1900-)) convention. The translation used is listed in the Bibliography.

8 Again, the subject can be understood as a claim (‘the agents are desire-realising’) with a subject (agents) and a predicate (being desire-realising).

9 “Analytic judgements could also be called *elucidatory*. For they do not through the predicate add anything to the concept of the subject; rather, they only dissect the concept breaking it up into its component concepts which had already been thought in it (although thought confusedly). Synthetic judgements, on the other hand, could also be called *expansive*. For they do add to the concept of the subject a predicate that had not been thought in that concept at all and could not have been extracted from it by any dissection.” (A7/B10-11)

10 “It is true that one might at first think that the proposition 7 + 5 = 12 is a merely analytic one that follows, by the principle of contradiction, from the concept of a sum of seven and five. Yet if we look more closely, we find that the concept of the sum of 7 and 5 contains nothing more than the union of the two numbers into one; but [in thinking] that union we are not thinking in any way at all what that single number is that unites the two. In thinking merely that union of seven and five, I have by no means already thought the concept of twelve; and no matter how long I dissect the concept of such a possible sign, still I shall never find in it that twelve. We must go beyond these concepts and avail ourselves of the intuition corresponding to one of the two: e.g., our five fingers, or (as *Segner* does in his *Arithmetic*) five dots. In this way we must gradually add, to the concept of seven, the units of five given in intuition”. (B15) It should be noted that debates continue over synthetic or analytic nature of mathematical propositions.

11 See Robert Hanna’s “The Return of the Analytic-Synthetic Distinction” (2012; reprinted in 2015).

12 This, as we have seen, is the way Kant presents the relation between subject and predicate for analytic propositions. The advantage is that there is no longer any need to specify how the predicate is supposed to be part of the subject (thought in the subject by the utterer, presupposed by the dictionary definition, implicit in the relevant linguistic community, etc.). The disadvantage is that this way of presenting analytic propositions is not entirely accurate. The predicate is not really identical with the subject, although, if identical, the proposition would still be analytic; but I understand this relation of identity as follows: it is impossible for the predicate to be denied of the subject (in the case of affirmative propositions). I will present this as an implication of identity, but in the context of my argument it is more basic in the understanding of the relation of identity for analytic propositions. Again, I allow this circular move here in order to avoid some of the debates concerning the analytic/synthetic distinction.

13 “…this sets us a problem that no other animal has. It is the problem of the normative. For our capacity to turn our attention on to our own mental activities is also a capacity to distance ourselves from them, and call them into question”. (93)

14 I do not think this process needs to be self-reflective, in the sense of being explicitly self-conscious; in other words, it need not involve my awareness that it is *me* who is thinking about my desires and similar mental activities; but it can be self-reflective simply in the sense that I reflect on *my* mental activities.

15 The sense in which they are right may be a precise one for the agent, but I need not specify at this stage a particular sense – say, a moral sense or an all-things-considered sense.

16 As an illustration, consider the example of ethnologist and philosopher of culture Mircea Vulcanescu. He had been incarcerated for political reasons by the Romanian Communist regime in September 1946 in the Aiud Penitentiary. The conditions in prison were appalling and, with Vulcanescu, there were also other ill inmates (he himself had contracted tuberculosis, but was not allowed any treatment in spite of recommendations from the penitentiary’s doctors); one of the inmates was so ill, he could not stand, and had to lie down on very cold concrete floor. To help him, Vulcanescu set himself on the floor, so that his ill cell colleague would lie on his body, rather than directly on the floor. Not doing anything when someone was in need was perhaps incompatible with Vulcanescu’s personality to the point of self-sacrifice.

17 Two issues can be considered here and it might be worth distinguishing them explicitly. There is first the issue of the unconditionality of a normative requirement. Let us say that I enjoy drinking coffee and that I now feel a desire to drink coffee, which is based on the expectation of enjoyment. Given that I value drinking coffee, there is a normative requirement for me to bring about a situation where I drink coffee. One question here is whether all normative requirements are of this type, namely, contingent on some subjective factor, such as the preference for coffee, which is presumably based on the particular way I am made up physiologically, as well as on my particular circumstances. Let us now assume that there is at least an unconditional requirement, a requirement that is not conditional on subjective factors. For instance, Korsgaard thinks that there is an unconditional requirement that we are committed to some value. The second question is whether all normative requirements (conditional or unconditional) are requirements or duties to myself or whether there are also requirements and duties to others, where this question is not simply a question of whether I can conceive of such duties and requirements to others, but whether there are such justified standards. The question I discuss in relation to Korsgaard is the former, not the latter. When the sceptic doubts normativity, she doubts that there is anything that can obligate her unconditionally.

18 Of course, an obligation requires that a particular action be performed, a prohibition requires that we refrain from performing a specific action, whereas a permission asserts that there is no obligation either to perform or to refrain from performing a particular action. What is common to these three claims is that the claims to the performance of the actions, to the refraining from the performance and to the possibility of performing or not performing are *unconditional*. In other words, the claims are not dependent on a further subjective condition for their validity.

19 Again, as mentioned before (see n17), this ‘must’ refers to an unconditional requirement, which is derived from non-subjective grounds. To act, a person must be committed to some standard (a norm or value reflected by her identity or project) and, as we have seen (in Section 2) this necessary commitment to some standard is what Korsgaard considers to be our identity as human beings. When the fact that a person is so committed to her humanity is also valued by that person, Korsgaard says the person has moral identity.

20 Arruda in the present special issue examines reflectivity as an aspect of agency in Korsgaard’s constitutivism.

21 For instance, in Illies (2003) or Stern (2015).

22 Recall that Korsgaard would like to show that the sceptic should acknowledge a commitment to the unconditional standard of having some evaluative identity or, in other words, he should acknowledge his moral identity; she thinks she can show this through a transcendental argument, which would show that his moral identity is constitutive of agency. This commitment is not a demand, which we actually are subject to in virtue of our moral identity; it is a commitment she thinks we have good reason to hold. By contrast, the commitment to having some identity is a commitment, which we have insofar as we are human beings, yet, it does not specify any particular demand, which we should actually be subject to; instead, it questions any such demand through the need for reasons for action. This is in short the answer to Smith’s objection discussed in Section 2.

23 This deductive transcendental argument can easily be put in the form of a retorsive transcendental argument as follows:

1. The sceptical agent (a sceptical human being who acts) doubts practical normativity.
2. A necessary condition for the possibility of agency is a commitment to a norm.
3. Hence, agential doubt of normativity implies normativity.

24 Korsgaard talks here about *rational* action, whereas so far we have talked about mere *action*; this is because Korsgaard has pursued her argument in terms of mere action too: “To act from such a conception [of the right and the good] is to have a practical conception of your identity. […] [F]or if you do not allow yourself to be governed by any conception of your identity, then you have no reason to act […] Does it really matter whether we act as our humanity requires, whether we find some ways of identifying ourselves and stand by them? […] If you had no normative conception of your identity, you could have no reason for action, and because your consciousness is reflective, you could then not act at all. Since you cannot act without reasons […] you must value your own humanity, if you are to act at all”. (122–3). As we will see later in this paper, however, there may be a good reason for her introduction of the qualifying “rational”.

25 For some illustrations with very suggestive titles, see Burnyeat (1998) and Greco (2012).

26 So, there are two sceptical theses: one, according to which the starting point is contingent; the second, according to which the derivation from the starting point to the conclusion is contingent. I can only consider the first one in this paper; the second continues to be debated in the literature and I do not need to solve it here for the argument in this paper.

27 When I talk about ‘humanity’, in this context, I have in mind that in virtue of which we are human beings – in this case, acting. Thus, insofar as he is a human being, that is, insofar as he has humanity, a person necessarily acts.

28 I do not mean to suggest that the only alternative, or even that the suggestion, in Korsgaard, would be that action is analytically presupposed by ‘human being’; it is equally possible that some other notion is analytically contained by ‘human being’ and this notion is part of a synthetic a priori principle, in virtue of which human beings necessarily act. A similar claim seems suggested by Korsagaard’s earlier *The Sources of Normativity* (1996).

29 If we assume that, in order to act, we need to be committed to a standard, then my action (whether right or wrong) will indicate I am committed to that standard. Yet, it is unclear whether this is a genuine commitment. As we have seen, for Korsgaard, being committed to standard S is the result of reflective success: I find a reason, which is sufficiently convincing about the value of S. Yet, the fact that I need to be committed to some identity in order to act is not a standard about which I seem to have any choice and does not seem to require any commitment. Its unconditional character is the result of the necessity of acting and this necessity of acting is not understood in any normative sense. On this issue, see the article by Christoph Hanisch in this volume.

30 This can be read as an implication of what, according to Henry Allison, is Kant’s Reciprocity Thesis: “This is the claim that freedom of the will and the moral law are reciprocal concepts. Kant affirms this explicitly in both works [the *Groundwork* and the *Critique of Practical Reason*]; correlatively, he also insists in both works that, although the moral law (or, better, the bindingness of the moral law for all rational agents) expresses a synthetic a priori proposition, it would be analytic if freedom of the will were presupposed.” (1986: 394) Given that freedom of the will is the freedom of the pure practical reason, rational action in this sense is also free action.

31 It might seem that there is no is-ought fallacy committed at this point: it might simply be a matter of deriving a robust ‘ought’ from rational/normative features of actions. A clarificatory note is therefore apposite: the objection is correct for the usual sense of the is-ought fallacy, but I take the fallacy in a more general sense; consider some rational/normative features of actions – f_1_, f_2_, … f_n_; if they belong to actions which are rational, then, when I attribute them to a rational action, I describe what a rational action *is*; the issue of going from f_1_, f_2_, …f_n_ to a robust ought is the same as that of going from the way a rational action *is* to the way it *ought* to be, were it to be moral.

32 According to Kant, for limited rational agents like us, the moral law appears in imperatival form, as a command. Hence, for us, the moral law is a Categorical Imperative. I use capital letters for this expression to distinguish it from the maxims, which pass the test of Kant’s Categorical Imperative, maxims that Kant sometimes calls categorical imperatives too. Korsgaard draws this distinction differently, calling the “moral law” what Kant usually calls the Categorical Imperative and, the “categorical imperative” what Kant usually takes to be a maxim: “Any law is universal, but the argument I just gave doesn’t settle the question of the *domain* over which the law of the free will must range. And there are various possibilities here. If the law is the law of acting on the desire of the moment, then the agent will treat each desire as a reason, and her conduct will be that of a wanton. If the law ranges over the agent’s whole life, then the agent will be some sort of egoist”. (99)

33 In her later *Self-Constitution: Agency, Identity and Integrity* (2009), Korsgaard no longer uses explicitly the idea of a transcendental argument, but can still be interpreted as formulating one (see, for instance, Guyer 2013). Her strategy, this time, is to introduce an additional argumentative step concerning self-constitution. Being committed to the Categorical Imperative is not simply a necessary condition for the possibility of good agency, but a necessary condition for the possibility of good self-constitution: “a commitment to the moral law is built right into the activity that, by virtue of being human, we are necessarily engaged in: the activity of making something of ourselves.” (2009: xiii)

34 Any moral theorist who is not a Kantian and who thinks that to act morally is to act rationally will challenge CA_2_”.

35 This is an example inspired by Peter Steinberger’s “The Standard View of the Categorical Imperative” (1999: 95)

36 There seems to be no reason to think that there would be a third possibility for accounting for necessity that would be introduced by Korsgaard; she does draw a distinction in her *Self-Constitution* between three forms of necessity, but there is no explicit account of how we are to understand the notion of necessity she favours. See Korsgaard (2009: 1–2).

37 See n10 above.

38 See also Baiasu (2011).

39 For a recent good discussion of some versions of transcendental idealism, see Richard Aquila (2015).

40 See also my texts Baiasu (2013, 2016).
